# Novel subsets of peripheral immune cells associated with promoting stroke recovery in mice

**DOI:** 10.1111/cns.14518

**Published:** 2023-10-31

**Authors:** Yichen Gu, Xiaotao Zhang, Huaming Li, Rui Wang, Chenghao Jin, Junjie Wang, Ziyang Jin, Jianan Lu, Chenhan Ling, Fangjie Shao, Jianmin Zhang, Ligen Shi

**Affiliations:** ^1^ Department of Neurosurgery Second Affiliated Hospital School of Medicine Zhejiang University Hangzhou Zhejiang China; ^2^ Clinical Research Center for Neurological Diseases of Zhejiang Province Hangzhou China; ^3^ Brain Research Institute Zhejiang University Hangzhou Zhejiang China; ^4^ Collaborative Innovation Center for Brain Science Zhejiang University Hangzhou Zhejiang China

**Keywords:** brain recovery, mouse model, peripheral immune cells, single‐cell RNA sequencing, stroke

## Abstract

**Aims:**

Peripheral immune cells infiltrating into the brain trigger neuroinflammation after an ischemic stroke. Partial immune cells reprogram their function for neural repair. Which immune cells promote ischemic brain recovery needs further identification.

**Methods:**

We performed single‐cell transcriptomic profiling of CD45^high^ immune cells isolated from the ischemic hemisphere at subacute (5 days) and chronic (14 days) stages after ischemic stroke.

**Results:**

A subset of phagocytic macrophages was associated with neuron projection regeneration and tissue remodeling. We also identified a unique type of T cells with highly expressed macrophage markers, including *C1q*, *Apoe*, *Hexb*, and *Fcer1g*, which showed high abilities in tissue remodeling, myelination regulation, wound healing, and anti‐neuroinflammation. Moreover, natural killer cells decreased cytotoxicity and increased energy and metabolic function in the chronic stage after ischemic stroke. Two subgroups of neutrophils upregulated CCL signals to recruit peripheral immune cells and released CXCL2 to keep self‐recruiting at the chronic stage.

**Conclusions:**

We identified subsets of peripheral immune cells that may provide potential therapeutic targets for promoting poststroke recovery.

## Brief abstract

The role of peripheral immune cells in stroke recovery remains unclear. Gu et al. report a phagocytic subset of macrophages and a macrophage‐like subset of T cells enhancing brain repair, while natural killer cells decrease cytotoxicity and neutrophils keep cell recruitment. These identified immune components may provide potential therapeutic targets.

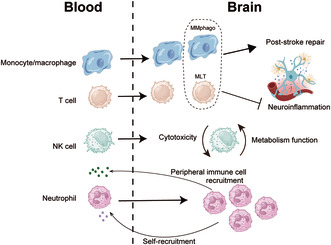

## 1 INTRODUCTION

Ischemic stroke is one of the leading causes of death and permanent disability worldwide.^1^ The nervous and immune systems orchestrate poststroke repair in a time‐dependent manner. Innate and adaptive immune cells produce immune‐modulating molecules and neurotrophic factors facilitating the reparative process, including myelin debris scavenging, remyelination, angiogenesis, extracellular matrix trimming, and neural circuit reconstruction.^2^, ^3^, ^4^ However, indiscriminate or overactive inflammatory responses in the global brain cause a secondary insult to neurons,^5^ hindering tissue restoration or neurotrophic effect. Thus, an optimal immune microenvironment is essential for sufficient and sustainable recovery after an ischemic stroke.

The peripheral immune cells reprogram themselves after infiltrating the ischemic brain and function together with the microglia.^6^, ^7^ Macrophages become inflammatogenic after crossing the blood‐brain barrier (BBB),^8^, ^9^ but gradually turn to a reparative state three to six days after stroke.^10^, ^11^, ^12^ T cells exacerbate neuron injury with IFNγ or neurotoxins,^13^, ^14^ while some CD4+ or CD8+ regulatory subsets help reduce infarct volume.^15^, ^16^, ^17^ Studies on the transcriptome level further identified differential subclusters, including resting, pro‐inflammatory, or neuroprotective components.^18^, ^19^, ^20^ These findings shed light on the complexity of neuro‐immune crosstalk after stroke. A comprehensive knowledge of the components and their dynamics is warranted to harness the poststroke immune response and improve poststroke outcomes.

We previously deployed single‐cell RNA sequencing on ischemic mouse brains at 5 days (5d) or 14 days (14d) after experimental stroke.^21^ In this study, we further identified two novel subpopulations of infiltrating peripheral immune cells that promote recovery: a GPNMB+ macrophage subset and a cluster of T cells expressing macrophage markers. Additionally, our results revealed decreasing cytotoxicity and increasing energy metabolic function in natural killer cells over time. Finally, two subgroups of neutrophils recruited peripheral immune cells via the CCL axis while keeping self‐recruiting via the CXCL axis during the recovery phase. These findings might provide potential therapeutic targets to facilitate recovery after stroke.

## 2 MATERIALS AND METHODS

### 2.1 Animals

Young (8–10 weeks old) male C57BL/6 mice were purchased from SLAC Laboratory Animal Company Limited (Shanghai, China). Mice were acclimatized to a 12/12‐h light/dark cycle. Food and water were provided ad libitum. All animal experiment protocols were approved by the Institutional Ethics Committee of the Second Affiliated Hospital, Zhejiang University School of Medicine, and complied with the Guide for the Care and Use of Laboratory Animals of the National Institutes of Health.

### 2.2 Murine models of transient cerebral ischemia

Transient cerebral ischemia (tMCAO) was induced by intraluminal occlusion of the left middle cerebral artery (MCA) for 60 min, as described previously.^2^ Sham‐operated animals underwent the same anesthesia and exposure of arteries without MCA occlusion. Briefly, mice were anesthetized, and an 8–0 monofilament with a silicon‐coated tip was introduced into the common carotid artery, advanced to the origin of the MCA, and left in place to limit MCA blood flow for 60 min.

### 2.3 Flow cytometry

Flow cytometry was performed to select CD45^high^ cells, as described previously.^21^ Briefly, animals were euthanized and perfused with cold saline. Brains were dissected to collect the ipsilateral and contralateral hemispheres. Brain homogenates were prepared with the Neural Tissue Dissociation Kit (T) (Miltenyi Biotec) following the manufacturer's instructions. Single‐cell samples were kept in the dark for 30 min at 4°C with primary antibody anti‐CD45‐PB (Biolegend, 103,126, 1:200), Single‐stained UltraCompeBeads were used for fluorochrome compensation. Flow cytometry was performed on the Beckman flow cytometer (Beckman CytoFlex). Data was analyzed on FlowJo software.

### 2.4 Immunofluorescence of brain sections

Brain samples were coronally cut into 25‐μm‐thick slices. Multiplex immunohistochemistry (mIHC) was performed by Opal Polaris 7‐Color Manual IHC Detection Kit (Akoya Biosciences, NEL861001KT), following the manufacturer's instructions. The following primary antibodies were used: anti‐CD8α (Abcam, ab217344, 1:100), anti‐C1Q (Abcam, ab182451, 1:100), and anti‐APOE (Abcam, ab183596, 1:100). Sections were observed with the automated imaging system Vectra® 3.0 (Akoya Biosciences).

Immunofluorescence staining was performed as previously described.^2^ Briefly, after blockage with 5% BSA in 0.3% triton X‐100 for 1 h at room temperature, the brain sections were incubated overnight at 4°C with the following primary antibodies: anti‐GPNMB (Abcam, ab188222, 1:250), anti‐MHCII (Thermo Fisher, 14–5321‐85, 1:100), and anti‐IBA1 (Abcam, ab5076, 1:200). Then the sections were incubated with the following secondary antibodies: donkey anti‐rabbit Alexa Fluor 647, (Invitrogen, 1:500), donkey anti‐rat Alexa Fluor 488, (Invitrogen, 1:500), donkey anti‐goat Alexa Fluor 555 (Invitrogen, 1:500). A Leica DM5500 microscope was used to observe the sections and capture images.

### 2.5 Single‐cell sequencing

Single‐cell sequencing was carried out in our previous study,^21^ and partial sequencing data was uploaded to the NIH Gene Expression Omnibus (GEO) database. The Access number is GSE171171. Briefly, ischemic brain hemispheres were perfused with cold saline at 5 and 14 days after tMCAO. Single‐cell suspension was generated for flow cytometry as previously described. CD45^high^ cells were sorted and a viability check was done by Cellometer Auto 2000 Automatic Cell Viability Counter system (Nexcelom). Live cells with >85% viability were used for library preparation. The Chromium instrument (10X Genomics) separated cells into gel beads in emulsion (GEMs). Reverse transcription was performed on the reaction mixture and emulsion with captured and barcoded mRNAs. cDNA samples were thus harvested and further fragmented and amplified. Finally, the libraries were sequenced on an Illumina HiSeq.

### 2.6 Basic analysis of single‐cell RNA sequencing data

The single‐cell RNA sequencing (scRNA‐seq) data was preprocessed by Cell Ranger version 3.0.1 (10X Genomics) and aligned to the GRCm38 (mm10) reference genome. Fundamental analyses were performed in R(v4.1.3) with the Seurat package (v4.3.0).^22^ Briefly, cells were included with detected genes ranging from 350 to 5500 and a mitochondrial gene ratio of less than 5%. Data normalization and variance stabilization were carried out with the SCTransform function. After principal component analysis (PCA), the FindClusters function was applied to identify different clusters, followed by nonlinear dimensional reduction methods used for visualization, including uniform manifold approximation and projection (UMAP) and t‐distributed stochastic neighbor embedding (tSNE).

### 2.7 Differentially expressed gene

Differentially expressed genes (DEGs) between different subclusters were identified with FindAllMarkers or FindMarkers functions. Wilcoxon rank sum tests were used with Bonferroni adjusted *p*‐value. Only genes with *p*‐value <0.01 and log_2_(fold change) >0.25 or < −0.25 were considered DEGs.

### 2.8 Gene ontology enrichment analysis

Gene Ontology (GO) enrichment analyses were performed on Metascape (http://metascape.org). A list of DEGs was uploaded and statistically enriched GO terms were returned. *Z*‐score was calculated with GOplot package in R to predict the activation state for each GO term.^23^ A term was considered to be significantly changed with *Z*‐score >2 or < −2 and adjusted *p* value <0.01.

### 2.9 Calculation of functional gene set module score

To quantify biological functions, the AddModuleScore method was applied to calculate the average expression levels of each specific gene set on a single cell level, subtracted by the aggregated expression of control gene sets. All analyzed genes are binned based on averaged expression, and the control genes are randomly selected from each bin. The gene sets were constructed based on the MSigDB molecular signature database (https://www.gsea‐msigdb.org/gsea/msigdb/index.jsp) and were listed in Table S6.

### 2.10 Cell–cell interaction analysis

The CellChat (v1.5.0)^24^ R package was applied to infer intercellular communication. Briefly, DEGs calculated as previously described were loaded and the official workflows were run in the R tool. The probability of interactions within each cluster was calculated and further grouped by signaling types.

### 2.11 Pseudotime analysis

The Monocle (v2.22.0) and Monocle3 (v.1.0.0) R packages were applied for pseudo‐time analysis.^25^, ^26^ Briefly, after inputting the Seurat object with clustering information, high‐variable genes were calculated, and those with a *p*‐value <0.01 and log_2_ (fold change) >0.25 were selected as ordering genes. The dimensional reduction was done with the DDRTree algorithm. The cellular trajectory was generated using the order_cells function and the plot_cell_trajectory function. Trajectories were plotted on UMAP coordinates in Monocle3. The pseudo‐temporal order of cells was constructed after choosing the starting point of the cellular trajectory. The one‐dimensional pseudo‐temporal distribution of cells was plotted with density function.

### 2.12 SCENIC analysis

SCENIC analysis was performed to explore different transcription factors (TFs) and their activities (regulon AUC) as previously described.^27^ The pySCENIC package (v.0.11.2) was used as a lightning‐fast Python implementation of the SCENIC pipeline. GRNBoost was applied to run the co‐expression modules. Gene‐motif rankings (10 kb around the transcription start site [TSS]) were used to determine the search space around the TSS for RcisTarget. The heatmap of normalized regulon AUC was plotted in R.

### 2.13 Statistical analysis

All statistical analysis methods were described in the figure legends. A comparison of the means between the two groups was made with the student's *t*‐test or the Wilcoxon rank sum test (both two‐tailed). Shapiro‐Wilk's method is used for the normality test. All statistical analyses were performed with R (v4.1.3) or GraphPad Prism (v.8.4.3). Statistical significance was defined as a *p*‐value <0.05.

## 3 RESULTS

### 3.1 Single‐cell profiling of immune cell infiltration in mouse brain after ischemic stroke

**FIGURE 1:**
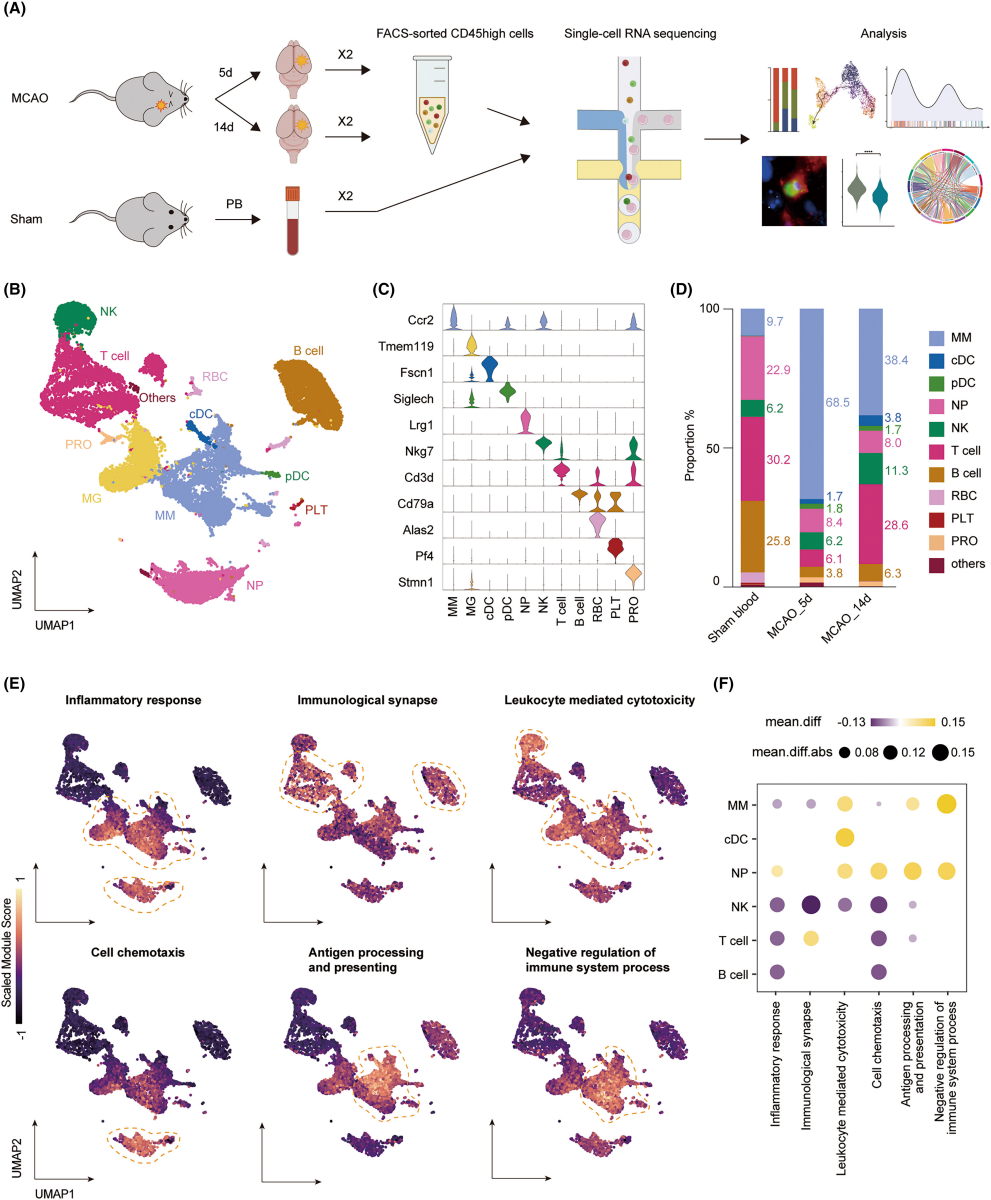
Single‐cell profiling of the immune microenvironment after ischemia. (A) The experimental scheme for single‐cell landscape dissection of ischemic brain and sham blood samples. (B) UMAP projection of cells harvested from the brain and blood. (C) Violin plot showing the selected marker genes of each cluster. (D) Stacked bar plot illustrating proportions of sequenced cells in the circulation and after infiltrating the brain at different time points. (E) Scaled module score based on functional gene sets. The dashed circles highlighted subclusters that were prominent in the current function. (F) Changes from 5 to 14 days of the mean module score for main cell types.

As described in our previous study, we carried out scRNA‐seq of CD45^high^ immune cells sorted from sham blood and the infarcted hemisphere on 5 and 14 days after transient middle cerebral artery occlusion (tMCAO) (Figure 1A). We chose time point 5 days as a representation of the subacute stage after stroke, which has prominent immune cell infiltration and neuroinflammatory response.^28^ Later, on approximately 14 days, the repair processes are known to initiate, and the brain enters a recovery phase.^17^, ^29^ We sequenced 26,832 cells from four ischemic brain hemispheres and two blood samples from the sham group. With quality control, 21,574 cells were chosen for downstream analysis.

We identified 12 cell types based on their enriched genes (Figure 1B,C, Table S1), including microglia (MG), mono‐macrophage (MM), conventional dendritic cell (cDC), plasmacytoid dendritic cell (pDC), neutrophil (NP), natural killer cell (NK), T cell, B cell, red blood cell (RBC), platelet (PLT), and proliferating cells (PRO). After immune cells entered the brain, myeloid cells were dominant on 5 days, accounting for 80.5% of all infiltrating cells, which in turn is dominated by MM, accounting for 68.5%. Later, on 14 days, the number of myeloid cells and lymphocytes reached a similar level (51.9% vs. 46.2%), with MM still predominating in the myeloid lineage (38.4%) and T cells in the lymphocytes (28.6%). Additionally, NK cell count increased during the recovery phase, and NP maintained its proportion around 8%, unlike the previous notion that NP decreased within one week after stroke,^28^, ^30^ Given the different cell compositions in circulation and the brain at the early or late phase, the infiltration of immune cells was likely to be a selective, time‐dependent process, suggesting unique and indispensable functions during the recovery period.

To explore the functions of each cell type, we calculated module scores based on functional gene sets in individual cells (Figure 1E, Figure S1A,B, Table S6). Infiltrating immune cells generally downregulated their inflammatory activity from 5 to 14 days, except neutrophils, which presented an increasing trend (Figure 1F). Macrophages were involved in the inflammatory response like a double‐edged sword, characterized by both pro‐inflammatory effects of antigen presentation and cytotoxicity as well as involvement in immune suppression. Their ability to reduce inflammation was noticeably more potent in the later stages indicating a critical role in long‐term recovery. We also saw a concurrent rise in cytotoxicity, suggesting that MM cells include a variety of heterogeneous components. Additionally, MM presented robust vesicle‐mediated intracellular transport implying a typical phagocytic function (Figure S1B). NP played an outstanding role in chemotaxis compared to other cells, with increasing strength. This function may be related to its stable cell proportion over time. Immunological synapses were mainly formed in T cells, indicating active interaction between T cells and their target cells, which may modify neural damage or repair. Meanwhile, this function was tuned upward in the recovery phase as well. Interestingly, we noted a decline in NK cytotoxicity but a rise in cell count, suggesting a phenotypical shift at the recovery stage. Therefore, further subgroup analysis of the above cells was crucial to understanding their diverse roles better.

### 3.2 A unique type of infiltrated macrophage reprogramed their transcriptome involved in phagocytosis and tissue regeneration

**FIGURE 2:**
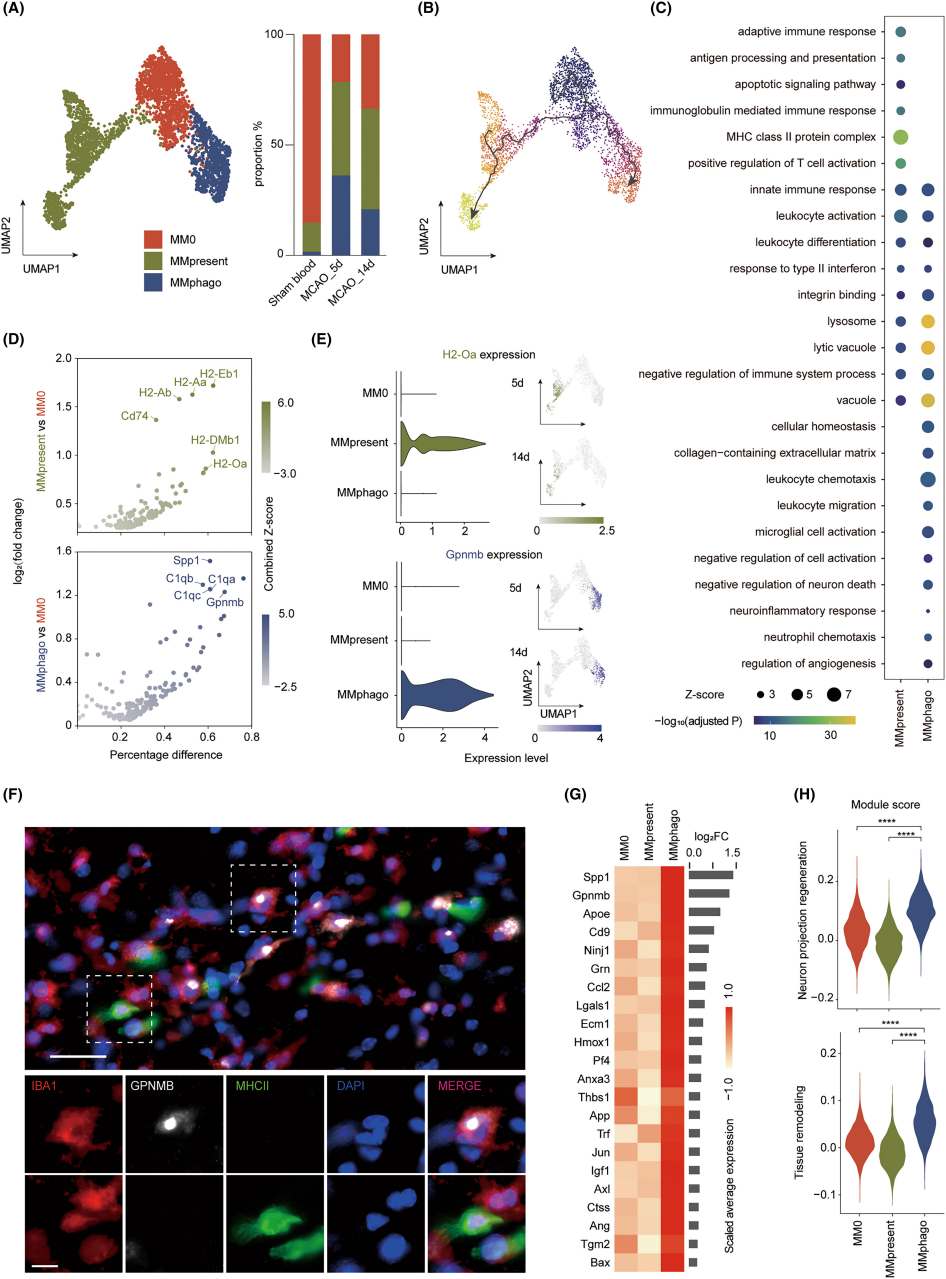
MMphago promoted tissue regeneration as one of the two MM phenotypes. (A) UMAP plot of all MM cells from sham blood and ischemic mouse brains after tMCAO, with each color representing one subcluster (left). And a stacked bar plot showing the proportion of the 3 clusters under each condition (right). (B) Pseudotime trajectory of the differentiation of MM analyzed with Monocle3, showing predicted shifting from MM0 (up) to MMpresent (lower left) or MMphago (lower right) based on the acquired cellular trajectory. (C) Dot plot illustrating representative functional terms of MMpresent and MMphago by GO enrichment based on *Z*‐score and significance (−log_10_[adjusted *p*‐value]). (D) Scatter plot showing the upregulated DEGs (log_2_(fold change) >0.25) of MMpresent or MMphago compared to MM0, with the percentage difference (defined as a percentage of the expression in MMpresent or MMphago – in MM0) along the *y*‐axis and log2 (fold change) along the *x*‐axis. The combined *Z*‐score of percentage difference and log2 (fold change) was shown in the color scale. (E) Violin plots and UMAP visualization of the marker gene *H2‐Oa* in MMpresent and *Gpnmb* in MMphago. (F) Representative images of GPNMB+ and MHCII+ MM immunofluorescence staining in the ischemic hemisphere on 5 days after MCAO. Scale bars, 30 μm (low magnification) and 10 μm (high magnification). (G) Heatmap showing the scaled average expression of reparative genes in each cluster (left), with bars showing log_2_ (fold change) of MMphago compared to MM0. (H) Violin plot displaying module scores of regeneration and remodeling functions. ****p< 0.0001 by the Wilcoxon rank sum test.

We subclustered and more deeply assessed the macrophage population. Four subsets (MM0‐MM3) were identified by unsupervised clustering (Figure S2A). The functions of MM1 covered those of MM3 revealed by GO enrichment, and both subclusters showed identical marker genes (Figure S2B,C). We, therefore, combined MM1 and MM3, dividing MM into three subpopulations. And two developmental directions were indicated by pseudo‐time analysis (Figure 2A,B). We noted that the subcluster MM0 lay at the beginning of the two differentiation directions and predominated in sham blood (85.2%). Monocyte‐associated genes (*Hp*, *Chil3*, *Ly6c2*) and early macrophage recruitment gene (*Ccr2*) were highly expressed in MM0 compared to the other MM cells (Table S2), indicating that MM0 may be in a resting state. Furthermore, we compared other MM subclusters to MM0. One direction favored antigen processing, presentation, and related major histocompatibility complex class II (MHCII) molecule function. The other showed a strong lytic effect, indicating an active phagocytosis phenotype (Figure 2C). We named them MMpresent or MMphago, respectively. From 5 to 14 days after stroke, MMpresent maintained its activity and proportion (42.4% on 5 days and 45.4% on 14 days) (Figure 2A, S2E). MMphago, although decreasing cell number from 36.1% to 20.8% (Figure 2A), experienced a slight upregulation of its overall functions, including lytic vacuole activity, leukocyte activation, regulation of cell death, and negative regulation of immune system process (Figure S2E).

Next, to verify these two types of MM, we screened the expression of typical markers of antigen presentation and phagocytosis. Compared to MM0, MHCII encoding genes (*H2‐Eb1*, *H2‐Aa*, *H2‐Ab*, *H2‐DMb*, and *H2‐Oa*) and helper gene (*Cd74*) showed high expression and superior specificity in MMpresent. Six of the top 10 DEGs were MHCII‐encoding genes. Meanwhile, MMphago differentially expressed complement coding genes (*C1qa*, *C1qb*, and *C1qc*), *Spp1*, and *Gpnmb* (Figure 2D, Tabel S2). *H2‐Oa* and *Gpnmb* showed consistent expression over time in MMpresent or MMphago (Figure 2E). Immunofluorescence staining verified MHCII+GPNMB‐ MMpresent cells and MHCII‐GPNMB+ MMphago cells in the peri‐infarct area both in subacute and recovery phases. (Figures 2F, S2D).

Macrophages participate in the late poststroke repair after phagocytosis.^10^, ^31^ *Spp1* and *Gpnmb* have been reported to be involved in cell‐matrix interaction or inflammation regulation respectively, two aspects of the repair process.^21^, ^32^ We found, apart from *Spp1* and *Gpnmb*, a group of repair‐related genes also elevated in MMphago on both 5 and 14 days (Figures 2G, S2F). We quantified MMphago's functions with module scores to investigate whether it was involved in late neural repair. Compared to other MM clusters, MMphago significantly contributed to neuron projection regeneration, tissue remodeling, and collagen formation (Figures 2H, S2G), which suggested that this subpopulation actively interacted with neurons and extracellular matrix after ischemic insult and may play a key effector role during the poststroke repair.

### 3.3 Macrophage‐like T cells exerted remodeling and anti‐inflammatory effects among lymphocytes

**FIGURE 3:**
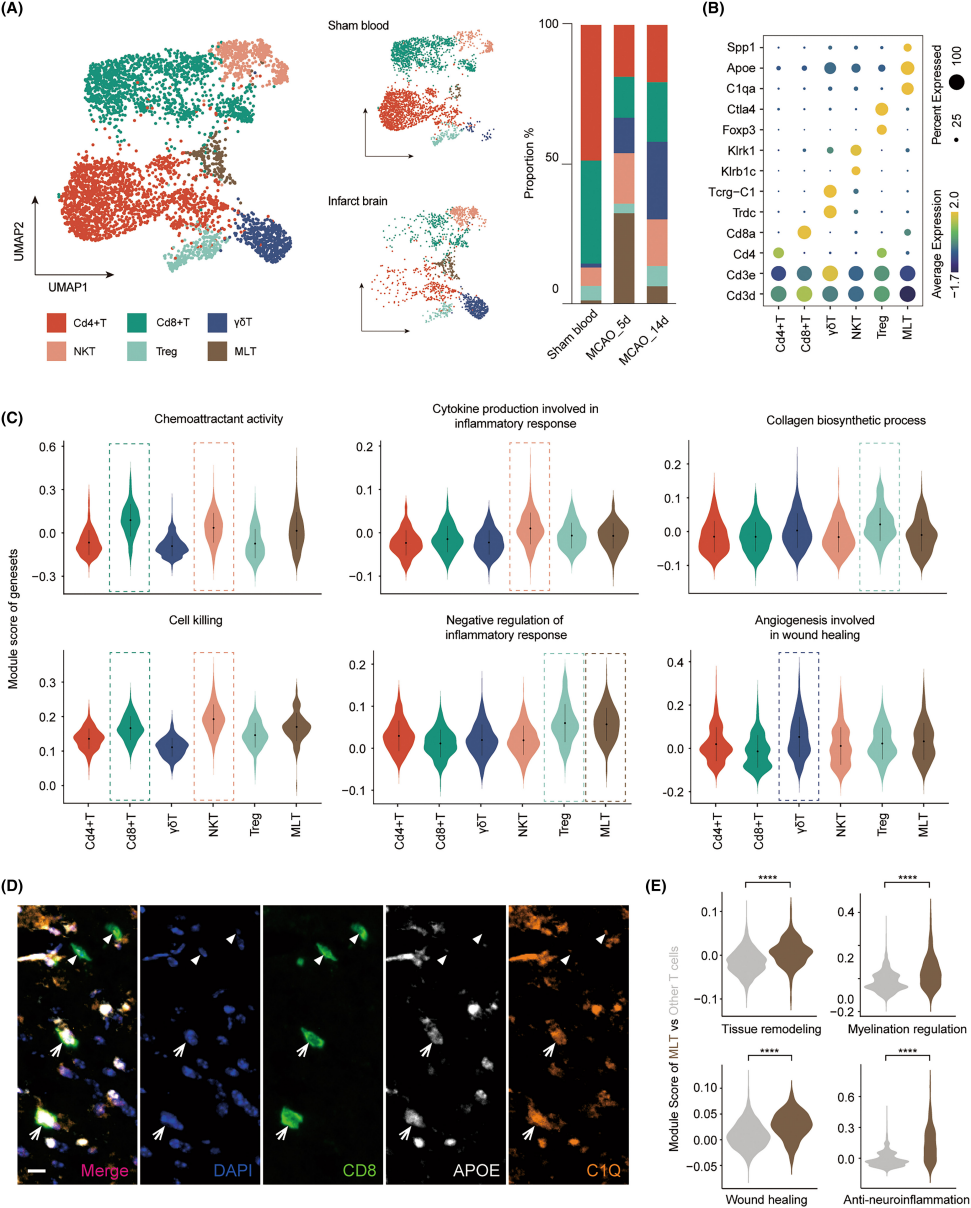
Macrophage‐like T cells exerted reparative effects among heterogenous T lymphocytes. (A) UMAP plot of T cells from different groups with aggregated visualization (left) or split by tissue (middle). Bar plots showing the proportions of the T cell subsets (right). (B) Dot plot of marker genes for the six T cell subclusters. (C) Violin plot of the module scores measuring characteristic functions of T cell subpopulations. The dashed rectangles highlighted clusters that were prominent in the current function. (D) Representative images of multiplex immunohistochemistry images of CD8 + APOE+C1Q+ MLT cells (arrow) and other CD8+ APOE‐C1Q‐ T cells (arrowhead) in the ischemic hemisphere on 14 days after MCAO. Scale bars, 10 μm. (E) Violin plot displaying module scores of reparative functions of MLT versus other T cells. ****p < 0.0001 by the Wilcoxon rank sum test.

We performed subpopulation analysis of T and B cells separately to investigate which cell subsets were beneficial for recovery. Six T cell subclusters, including Cd4 + T, Cd8 + T, regulatory T (Treg), gamma‐delta T (γδT), and natural‐killer T (NKT), were identified according to their typical marker genes (Figure 3A,B). Infiltration of peripheral T cells into the brain seemed to be an active and selective process, with the distribution of T cell subsets in the brain different from that in sham blood, where Cd4 + T and Cd8 + T cells were predominant (more than 80%). Infiltrating NKT and γδT in the brain had a remarkably higher proportion (18.1% and 12.7% on 5 days, respectively) than in the blood, and there was an increasing trend for γδT over time, reaching 27.8% on 14 days. Module score calculation revealed the characteristics of each cluster (Figure 3C). Cd8 + T was prominent in the chemotaxis with high expression of *Ccl5* and *Mif* (Figure S3A). NKT cells mainly participate in chemoattracting, cell killing, and inflammatory reactions. γδT cells had a greater promoting effect on blood vessel regeneration, expressing relatively higher *Gpx1*, *Tnf*, and *Xbp1* (Figures 3C, S3A), which suggested a potential pro‐repair role of γδT, unlike a solely detrimental one as previously believed.^33^ Treg negatively regulated the immune system and participated in the collagen biosynthesis process, consistent with previous findings.^15^, ^17^, ^21^ We did not perform a transcriptomic comparison between 5 and 14 days due to the vast difference in cell counts (Figure 1A).

Interestingly, we found a novel subpopulation of T cells expressing genes usually seen in macrophages, including *C1q*, *Apoe*, *Hexb*, and *Fcer1g* (Figure S3B). We named it macrophage‐like T (MLT). Considering expression and specificity, we chose C1Q and APOE as markers of MLT. Multiplex immunohistochemistry confirmed the existence of this subpopulation, which co‐expressed C1Q, APOE, and the T cell marker CD8 (Figure 3D). On 5d, very few T cells entered the brain, but MLT took up a considerable portion of 32.4% (Figure 3A). This timed accumulation of MLT on 5d motivated us to explore the biological functions. Module score quantification revealed a significantly stronger anti‐inflammatory effect as well as powerful abilities in tissue remodeling, myelination regulation, and wound healing (Figure 3E), with high expression of repair‐related genes (*Apoe*, *Trem2*, *Grn*, and *Igf1*) (Figure S3A, Table S3). To further explore the potential regulon activity, we identified *Mafb*, *Cebpa*, *Psmd12*, and *Irf5* as candidate TFs with specifically upregulated expression levels in MLTs (Figure S3C,D). These findings suggested that MLT, like MMphago, might also play an essential role in neural recovery.

B cells experienced a functional reprogram after entering the brain in that they enhanced activities in complement C1q complex, leukocyte differentiation, positive regulation of cell death, and maintenance of location in cell and multivesicular body, indicating a continuous immune activation and engulfing function (Figure S4A,B). Fifty‐two DEGs selectively upregulated on 5d were associated with the activation of BCR and metabolic changes in response to tissue injury. The sixty upregulated DEGs on 14 days were implicated in the presentation of extracellular peptides and protein synthesis. In general, B cells showed significantly higher antigen processing and presentation activity and immunoglobin production on 14 days (Figure S4C), two classical B lymphocyte functions.

### 3.4 Natural killer cells demonstrated decreasing cytotoxicity and increasing energy metabolic function at the chronic stage after ischemic stroke

**FIGURE 4:**
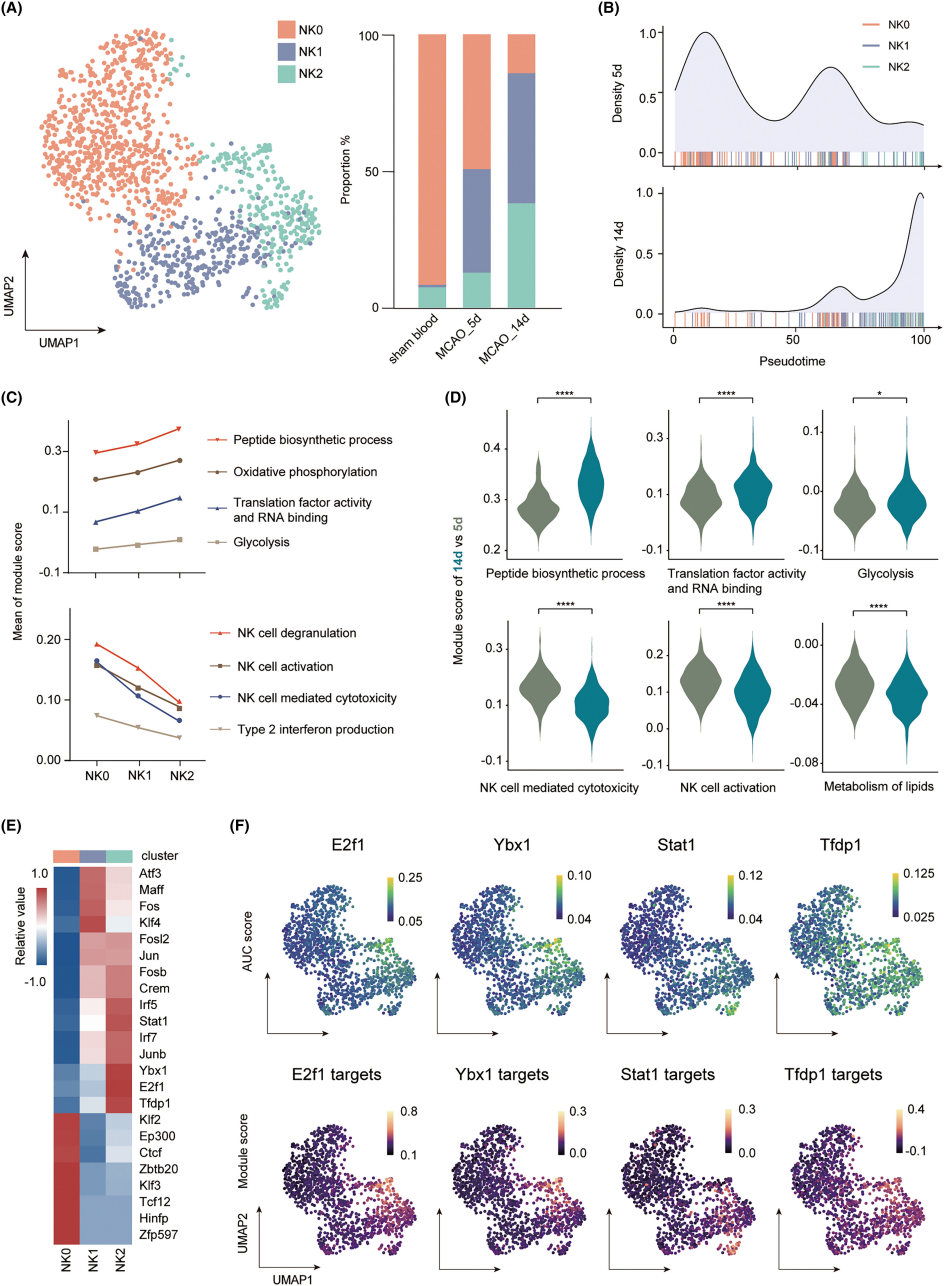
Natural killer cells demonstrated decreasing cytotoxicity and increasing energy metabolic functions over time. (A) UMAP plot of NK cells from sham blood and ischemic mouse brains after MCAO, with each color representing one subcluster (left). Stacked bar plot showing the proportion of the three clusters under each condition (right). (B) The distribution of NK cells on pseudo‐temporal axes in the ischemic brain. Color‐coded bars indicated the positions of NK cells on pseudo‐temporal axes. (C) Line chart displaying the functional differences among NK subsets, measured by module scores. (D) Violin plot showing functional dynamics from 5 to 14 days. *p < 0.05, ****p < 0.0001 by the Wilcoxon rank rum test. (E) Heatmap showing specific transcription factors of each cluster, with color scale representing relative expression. (F) Feature plot showing the expression level of NK2‐specific transcription factors measured by AUC score (up). And scaled module score based on the target gene list of each transcription factor (down).

Natural killer (NK) cells were divided into three clusters (Figure 4A, Table S4). NK0 accounted for 91.7% in sham blood but decreased continuously after stroke, accounting for 49.3% of all NK cells on 5 days and 14.3% on 14 days. Meanwhile, NK1 and NK2 grew in number, constituting 37.9% or 12.8% of all NK cells on 5 days and 47.6% or 38.2% on 14d, respectively. Pseudo‐time analysis revealed a linear, unidirectional evolution process from NK0 to NK2 (Figure S5A). We projected the distribution of NK cells on a pseudo‐temporal axis, unraveling a shift from an NK0/1 dominant pattern on 5 days to an NK1/2 dominant pattern on 14 days (Figure 4B). Interestingly, along with the pseudo‐temporal evolution from NK0 to NK2, NK cells kept attenuating their activation, cytotoxicity, degranulation, and type 2 interferon production. However, they showed increasingly higher activity in the peptide biosynthetic process, oxidative phosphorylation, glycolysis, and RNA binding by translation factors (Figure 4C). These alterations corresponded to the three main functions of NK2 in immune activity, metabolism, and biosynthesis (Figure S5B). In the actual chronological order from 5 to 14 days, NK cells also significantly enhanced biosynthetic and translation functions as well as glycolysis while downregulating their activation, classical cytotoxicity, and metabolism of lipids (Figure 4D). This phenotypic shift suggested a specific role for NK at different periods.

Liu et al. revealed transcription factor *Runx3* as a key role in maintaining proper NK cell function—dysregulation of *Runx3* mediated decreased NK response in ischemic brain.^34^ We also found a similar downregulation of *Runx3* in NK1 and NK2 (Figure S5C). SCENIC analysis was performed to further explore specific transcription factors (TFs) in each cluster. NK2 highly expresses *E2f1*, *Tfdp1*, *Stat1*, and *Ybx1*. The targets of these TFs were also enriched in NK2 (Figure 4E,F). *Ybx1* is usually reported in tumor migration and invasion, exerting its function by activating NF‐κB signaling.^35^ NF‐κB genes were also elevated in NK2 (Figure S5D). NK cells can be activated via their surface receptors or cytokines.^36^ Upregulated *Stat1*, *Irf5*, *Irf7*, and genes of cytokine receptors indicated that various cytokines provided activation signals to NK2 (Figure S5D). However, receptor signals were declining, with relatively low expression of activating receptors but high inhibitory ones (Figure S5D). This phenomenon may be due to phagocytic cells in the microenvironment, which cleaned up the myelin fragments or other debris capable of activating receptors on NK. Together, NK cells demonstrated an alteration over time with decreasing cytotoxicity and increasing energy metabolism.

### 3.5 Two highly‐activated subsets of neutrophils were identified to recruit immune cells at the recovery phase after ischemic stroke

Neutrophils (NP) were believed to be involved in the inflammatory response in the early stages of stroke and gradually decrease in the later stages.^28^, ^30^ Our data, however, found no significant decrease in the proportion of NP over time (Figure 1D). Subset analysis of NP revealed that NPblood was predominant, with a proportion of 87.5% in sham blood, expressing markers of circulating neutrophils like *Retnlg*, *S100a6*, and *Ccl6* ^37^ (Table S5). In the early poststroke period, there were five main states of neutrophils, namely preNP, NP1, NP2, NP3, and NPblood, while the proportion of NP2 and NP3 increased remarkably in the late poststroke period (23.3–56.5% for NP2, 3.1–17.3% for NP3).

**FIGURE 5:**
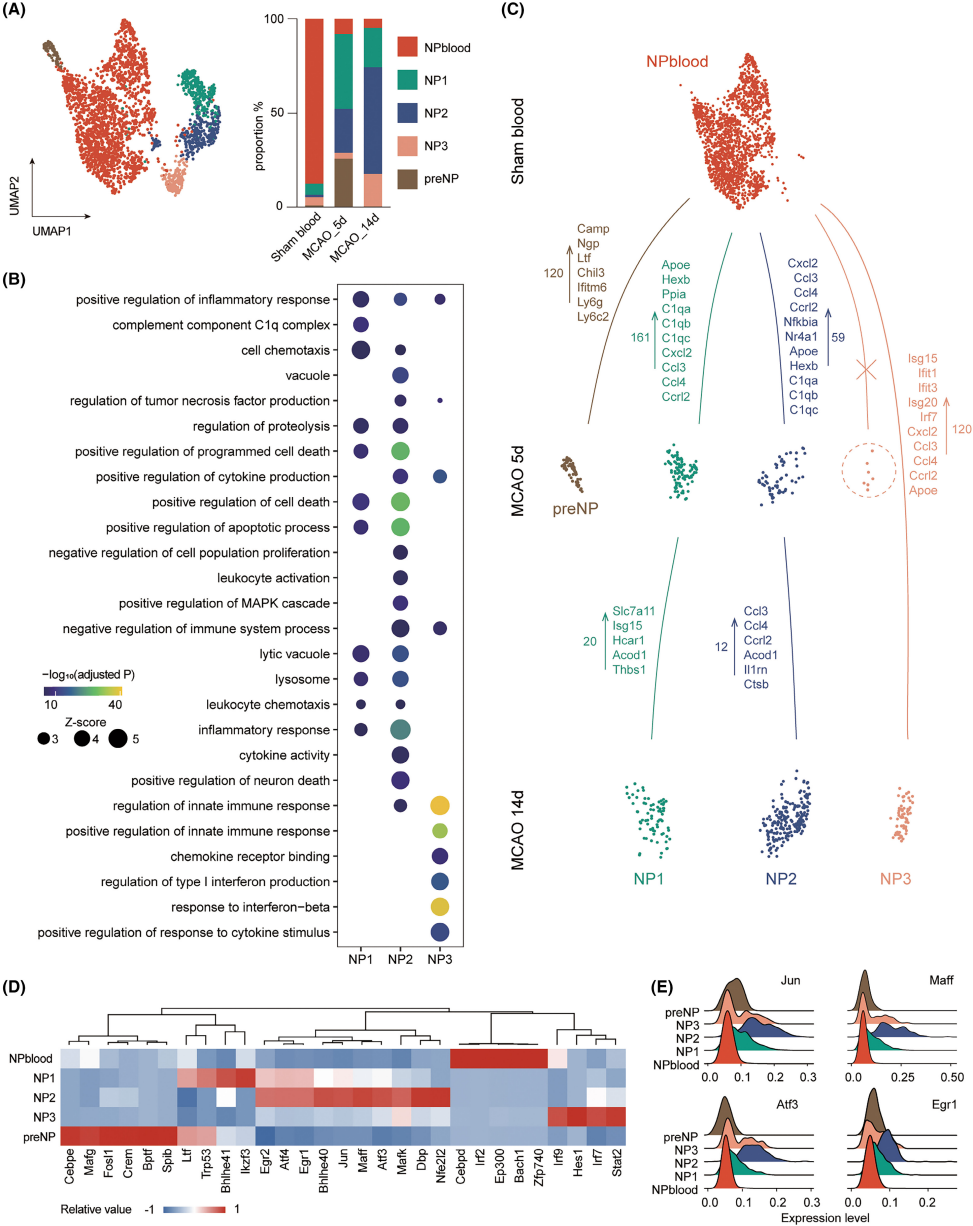
Chemotaxis and inflammatory functions of neutrophils during the subacute and recovery phase. (A) UMAP plot of NP cells from sham blood and ischemic mouse brains after MCAO, with each color representing one subpopulation (left). A stacked bar plot showing the proportion of NP subpopulations in each group (right). (B) Dot plot illustrating representative functional terms of NP1, NP2 and NP3 by GO enrichment based on *Z*‐score and significance (−log_10_[adjusted *p*‐value]). (C) Interpretation of the compositional transition of NP subpopulations over time after ischemic injury. Representative upregulated DEGs between two groups are listed, along with numbers. The cell count of NP3 on 5 days was too small for a biologically meaningful comparison. (D) Heatmap showing specific transcription factors of each NP cluster, with a color scale representing relative expression. (E) Ridge plot showing the expression level of NP2‐specific transcription factors measured by AUC score.

PreNP emerged in the early poststroke period and occupied 25.7% of NPs (Figure 5A). It highly expressed genes indicating a naïve stage (*Camp*, *Ltf*, *Ngp*, and *Chil3*) and participated in inflammatory responses on 5d (Figure 5C, S6A, B, Table S5). The presence of preNP in the brain may be due to stress‐induced hematopoiesis after an ischemic insult and may cause the rapid accumulation of NP in the subacute phase. NP1 played a significant role of 39.7% on 5 days, apart from preNP. It was involved in engulfing, inflammatory response, and positive regulation of cell death, with elevated genes related to complements (*Apoe*, *Hexb*, *C1qa*, *C1qb*, and *C1qc*) and chemotaxis (*Cxcl2*, *Ccl3*, *Ccl4*, and *Ccrl2*) (Figure 5B,C, S6C, Table S5). These findings suggested a neurotoxic role of NP1, consistent with conventional perceptions.^30^ Interestingly, in the recovery phase, NP2 and NP3 were significantly expanded, showing a robust inflammatory effect and chemotactic function, upregulating corresponding genes (*Tlr2*, *Ctsb*, *Il1b*, *Ccl3*, *Ccl4*, and *Ccrl2*) (Figure 5C, S6C, Table S5). Noteworthy, NP3 demonstrated a strong regulatory effect on intrinsic immunity as well as response to interferon (IFN), upregulating IFN‐related genes such as *Isg15*, *Ifit1*, *Ifit3*, *Isg20*, and *Irf7* (Figure 5B,C, Table S5). These two clusters outweighed NP1 and may play a unique recruiting role in the late phase.

To investigate the translational regulation of different NPs, we performed a SCENIC analysis that recognized TFs enriched in each cluster. NP1 highly expressed *Ikzf3* and *Bhlhe41*. *Jun*, *Maff*, *Atf3*, *Egr1*, and other TFs were enriched in NP2. Jun was a crucial participant in the inflammatory response of neutrophils and was reported to be an inducer of *Cxcl2*. Expression of *Jun* is mainly in the late neutrotime, indicating a mature state of neutrophils and prolonged activity.^37^, ^38^ *Maff*, *Atf3*, and *Egr1* were involved in inflammation or neutrophil migration.^39^, ^40^, ^41^ Among them, *Egr1* was also reported to induce *Atf3*.^42^ These TFs altogether contributed to the inflammatory and chemotactic traits of NP2. NP3 upregulated *Irf7*, *Irf9*, and *Stat2*, as TFs responding to interferons.^43^ The *Hes1* in NP3 was believed to attenuate inflammation and recruitment of neutrophils,^44^ which may explain a relatively weak inflammatory response in NP3 (Figure 5B). Additionally, preNP expressed *Cebpe*, known to mediate maturation and terminal differentiation of granulocytes (Figure 5D,E, and S6D).^45^

### 3.6 NP2 and NP3 were involved in chemotaxis, and MMphago received TGFβ signals

**FIGURE 6:**
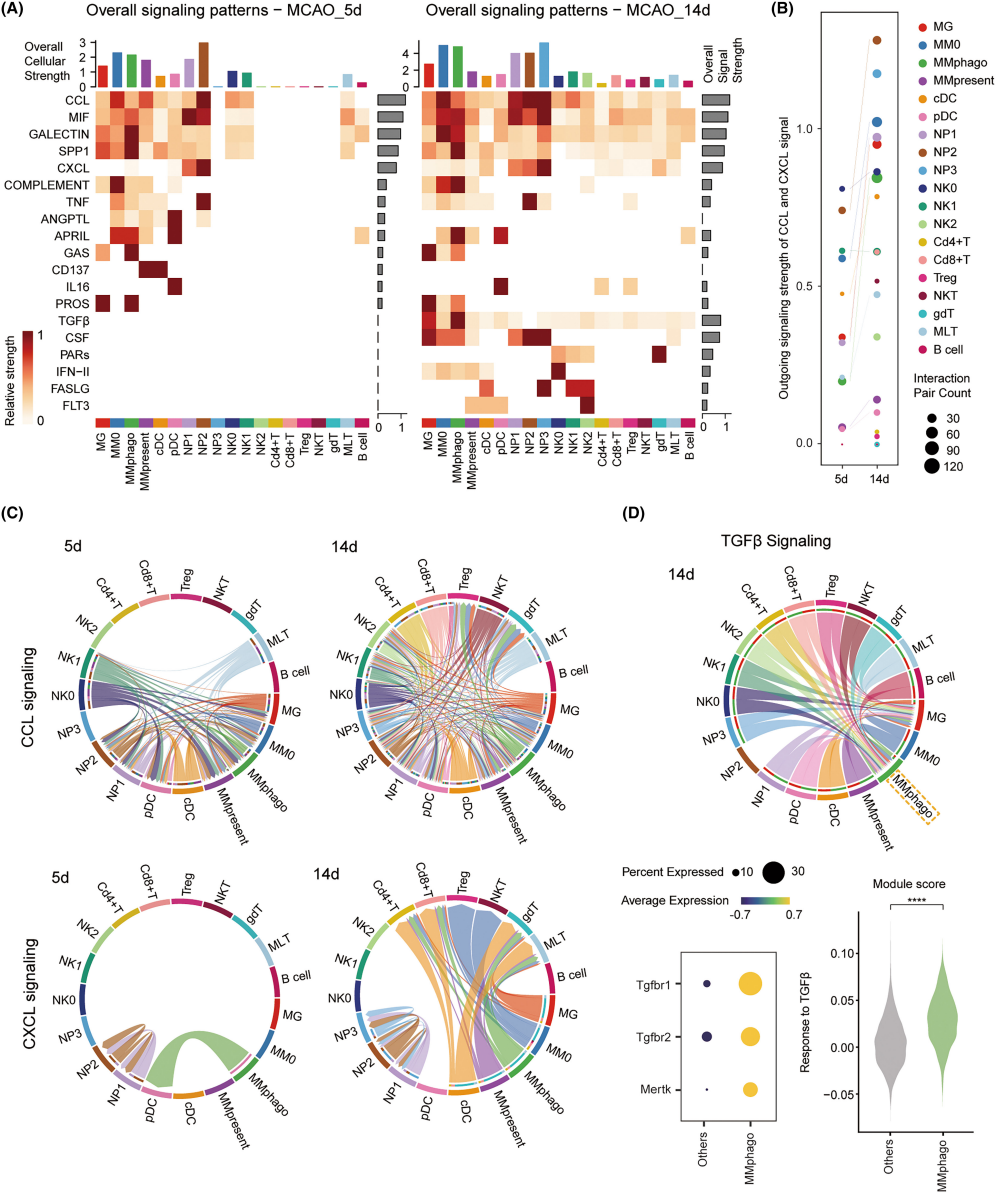
NP2 and NP3 were involved in chemotaxis, while MMphago received TGFβ signals on 14 days. (A) Heatmaps showing the relative strength of the overall signals among the interaction network of immune cells on 5 or 14 days, with a bar plot summarizing the total strength of individual clusters (up, Overall cellular strength) or signal pattern (right, Overall Signal Strength). (B) Bubble plot showing the strength of releasing CCL or CXCL signals from each cluster in the ischemic brain. (C) Chord plots of CCL or CXCL signaling network among the immune cells on 5 days (left) or 14 days (right). Arrows represent signals from the releasing cells to the receptor cells. (D) Chord plots of the TGFβ signaling network of MMphago and other immune cells on 14 days (up). Dot plot showing expressions of TGF receptor MERTK‐encoding genes (lower left). Violin plot illustrating TGFβ–responding function (lower right). ****p < 0.0001 by the Wilcoxon rank sum test.

Multiple cell types infiltrated into the brain and further interacted with each other dynamically, adding complexity to the microenvironment. To dissect this network, we applied CellChat developed by Jin et al.^24^ and revealed enhanced intracellular interactions over time. The CCL signal was dominant at both subacute and chronic phase. Meanwhile, NP was the most active participant, and mainly involved in CCL and CXCL signaling, remaining consistent on both 5 and 14 days (Figure 6A). From a combined chemoattracting perspective, NK and NP released the most chemotactic signals on 5 days. While on 14 days, NP greatly enhanced its effect and was far ahead of other cells, especially NP2 and NP3 (Figure 6B).

The CCL signal mainly recruited nonspecific immune cells like MMphago at 5d, compared to the signal flow of CXCL (Figure 6C (left), arrows representing signal from the releasing cells to receptor cells). Later, Treg and γδT were attracted along with MM by CCL signal (Figure 6C (right)). Given the pro‐repair potential of these three cell types, the CCL signal may be involved in constructing an immune milieu in favor of neural recovery, MMphago being a critical component. CXCL signal, however, was involved in recruiting specific immune cells. MLT was mainly attracted by CXCL molecules, while Treg and γδT responded to dual signals from CCL and CXCL family. Interestingly, NP demonstrated a self‐recruiting pattern with CXCL2‐CXCR2 signal throughout the disease (Figure 6C (down), Table S7), with the ever‐increasing chemotactic function of NP2.

Additionally, some new types of signaling pathways emerged on 14d, including TGFβ (Tgfb‐Tgfbr1/2), CSF (Csf1‐Csf1r), PARs (Gzma‐F2r), IFN‐II (Ifng‐Ifngr1/2), FASLG (Fasl–Fas), and FLT3 (Flt3l‐Flt3) (Figure 6A, Table S7). MMphago received TGFβ signals from various types of cells. It also upregulated the corresponding receptors *Tgfbr1* and *Tgfbr2* and functionally enhanced the downstream response to TGFβ. TGFβ was reported to assist macrophages in recruiting^46^ and enhance their phagocytosis and the ability to clean up myelin debris by upregulating a core tyrosine kinase *Mertk*.^31^ We also identified elevated expression of *Mertk* in MMphago (Figure 6D).

## 4 DISCUSSION

We leveraged scRNA‐seq in mouse brains 5 or 14 days after stroke to dissect immunological components and dynamics in poststroke repair. We identified cell clusters exerting recovery functions: MMphago associated with neuron projection regeneration and tissue remodeling, and MLT involved in tissue remodeling and anti‐neuroinflammation. NK cells decreased their cytotoxicity with an increasing energy metabolic function over time. Two highly activated NP subsets kept recruiting peripheral immune cells, including NP cells themselves, in the recovery phase.

The innate immune response, represented by the infiltration and activation of monocytes and macrophages (MM), is one of the main events in the acute inflammation of stroke.^8^ The proportion of infiltrating MM dropped from 68.5% on 5 days to 38.4% on 14 days. This decrease was reported to start approximately one week after the stroke.^28^ Moreover, MM functionally shifted from a pro‐inflammatory state to a repairing one within three to six days.^10^, ^11^ We identified two developing directions to phagocytosis or antigen presentation of infiltrating MM. The MMphago played a reparative role, releasing trophic factors for tissue restoration and circuit reorganization,^12^ and resolving inflammation by clearing inflammatory substances in postischemic tissue. It specifically expresses the phagocytosis‐related molecules *Mafb* and *Mertk* (Figure 6D, Table S2).^10^, ^31^ MMphago received TGFβ signal from multiple sources in the immune microenvironment, which may contribute to its phagocytic and reparative phenotype. Noteworthy, the classification of M1/M2 has been frequently mentioned but has also been criticized for the lack of specificity.^47^ Though the M2 phenotype was alleged to be a reparative pole in macrophage development, we find that MMphago differentially expressed marker genes of both M1 (*Fcgr1* and *Fcgr3*) and M2 (*Il10r*, *Arg1*, and *Cd68*) (Table S2). Thus, the phagocytosis/presenting phenotypes may be more suitable for describing poststroke macrophage dynamics.

T cell infiltration came after myeloid cells with a higher proportion on 14d (Figure 1D), participating in inflammation or brain repair. As an immunosuppressive subtype, Treg plays a neuroprotective role and has been a potential target for poststroke recovery, reducing infarct volume, inhibiting gliosis, or regulating the reparative function of other cells.^48^, ^49^ Here, we report MLT as another T cell subset with reparative potential. These cells are debris scavengers like macrophages or microglia and promote tissue regeneration (Figure 3E). Unlike Treg, which accumulated at the recovery stage, MLT occupied a considerable proportion on 5 days (Figure 3A). This suggested that MLT may play a major role in T cell‐mediated repair during the subacute phase, while Treg involved in a late‐stage repair. The γδT has been believed to be an inflammation mediator that releases IL‐17 to propagate ischemic injury. However, moderate inflammation can also promote the regeneration of blood vessels, alleviating ischemia and promoting tissue repair.^33^, ^50^ In addition, γδT is also easy to regulate, for example, there is a close connection between gut microbiota and γδT.^51^ How to regulate γδT into a pro‐repair phenotype will be a future research direction. Generally, T cells infiltrated less on 5 days and did not occupy a significant proportion of the brain until the recovery phase.^52^ CXCL chemotaxis may be pivotal in attracting T cells, which became apparent at 14 days. The signal was mainly released by MM and DC (Figure 6C). However, it was reported that T cell accumulation could last for 1 month after stroke onset in mice and even 140 days in humans, probably due to local proliferation rather than infiltration.^53^, ^54^ The exact origins and repair‐related functions of T cells in the brain during a longer‐term period remain to be elucidated.

Neutrophils are the first immune cells to enter the lesion. They release pro‐inflammatory cytokines like IL‐1b and neutrophil extracellular traps (NETs) to mediate a secondary brain injury after stroke (Figure 1E, S5C).^30^, ^55^ The proportion of infiltrating neutrophils remained stable, which could partially be explained by the self‐recruitment via CXCL2–CXCR2 interaction, allowing neutrophils to enter the brain during the recovery phase (Figure 1D, 6C, Table S7). Meanwhile, CXCL2 released by neutrophils can also target endothelial cell junctions, enhancing the diapedesis across BBB, and further propagating neutrophil infiltration.^56^ Inhibiting the progressive process of self‐recruitment might be beneficial. Additionally, in attempts to optimize the microenvironment for repairing, immune modulation of the periphery may also be necessary since there were still immune cells infiltrating the brain during the recovery period.

The present study has the following limitations: First, we only focused on immune cells infiltrating the brain. Microglia, as resident immune cells of the brain, orchestrate acute inflammation at the early stage but exert phagocytic and neurotrophic functions in the late phase, which has been extensively studied.^6^, ^7^, ^57^ The interactions of microglia with infiltrating immune cells need further study. Second, the repair requires concerted efforts of immune cells and nonimmune cells, including astrocytes, oligodendrocytes, endothelial cells, and neurons. Neurons are fragile to shear stress during single‐cell suspension preparation, making it difficult to acquire their transcriptome simultaneously with immune cells. Other sequencing techniques, like single‐nuclear sequencing, may help create a thorough landscape of poststroke repair. Third, the current study analyzed the subacute phase (5d) and the starting point of recovery (14 days) after stroke. The acute or super‐acute phase includes initial microglia mobilization and infiltration of neutrophils, while in the late phase after 1 month, T cell proliferation can be sustained in the brain. Studies on a broader time scale are warranted.

## 5 CONCLUSIONS

In conclusion, we identified MMphago and MLT, two novel subsets of infiltrating peripheral immune cells in the mice brain, which promoted the repair process after stroke. These findings shed new light on potential therapeutic targets for poststroke recovery.

## AUTHOR CONTRIBUTIONS

Yichen Gu and Xiaotao Zhang contributed equally to this work. Ligen Shi and Jianmin Zhang designed the research. Yichen Gu, Xiaotao Zhang., Huaming Li, Rui Wang, Chenghao Jin, Ziyang Jin, Jianan Lu, Chenhan Ling, and Fangjie Shao performed the experiments. Yichen Gu and Xiaotao Zhang analyzed and/or interpreted the data. Yichen Gu, Xiaotao Zhang, and Rui Wang wrote the paper. Ligen Shi and Jianmin Zhang critically revised the paper. Junjie Wang modified the language.

## CONFLICT OF INTEREST STATEMENT

The authors declare that they have no competing interests.

## Supporting information


**Figures S1–S6** **.**

**Tables S1–S6** **.**

## Data Availability

The data that support the findings of this study are available from the corresponding author upon reasonable request.
