# Supplementary material for: Novel subsets of peripheral immune cells associated with promoting stroke recovery in mice
Source: CNS Neurosci Ther. 2023 Oct 31;30(4):e14518. doi: 10.1111/cns.14518 (PMC11017448; doi:10.1111/cns.14518)
Supplement: Supplementary file 1 — Figures S1–S6 . [file CNS-30-e14518-s001.docx]

**Supplementary Information for**

**Novel Subsets of Peripheral Immune Cells Associated with Promoting Stroke Recovery in Mice**

Yichen Gu, Xiaotao Zhang, Huaming Li, Rui Wang, Chenghao Jin, Junjie Wang, Ziyang Jin, Jianan Lu, Chenhan Ling, Fangjie Shao, Jianmin Zhang, and Ligen Shi.

^#^ To whom correspondence should be addressed:

Dr. Ligen Shi, Department of Neurosurgery, Second Affiliated Hospital, School of Medicine, Zhejiang University, 88 Jiefang Road, Hangzhou, Zhejiang 310009, China. Tel: +86-571-87784715; Fax: +86-571-87784755; email: [slg0904@zju.edu.cn](mailto:slg0904@zju.edu.cn).

Dr. Jianmin Zhang, Department of Neurosurgery, Second Affiliated Hospital, School of Medicine, Zhejiang University, 88 Jiefang Road, Hangzhou, Zhejiang 310009, China. Tel: +86-571-87784715; Fax: +86-571-87784755; email: [zjm135@zju.edu.cn](mailto:zjm135@zju.edu.cn).

**This file includes:**

**Supplementary Figures 1-6**

**
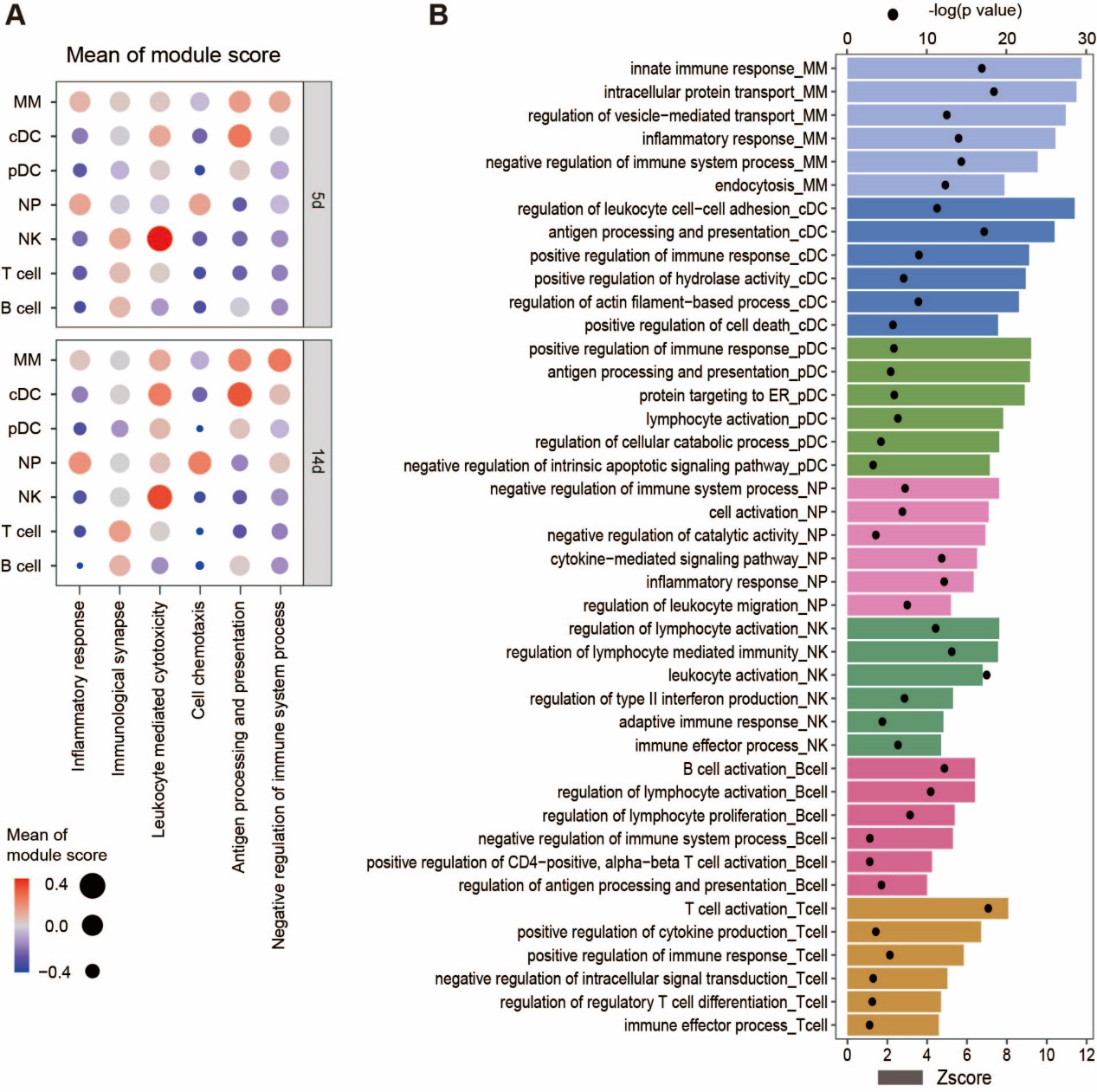
**

**Figure S1. Function analysis of infiltrating immune cell clusters.** (A) The mean value of the scaled module score in each cluster, split by 5d or 14d. (B) Bar plot showing the major upregulated gene ontology (GO) terms based on Z-score and significance (-log_10_[adjusted P-value]).

**
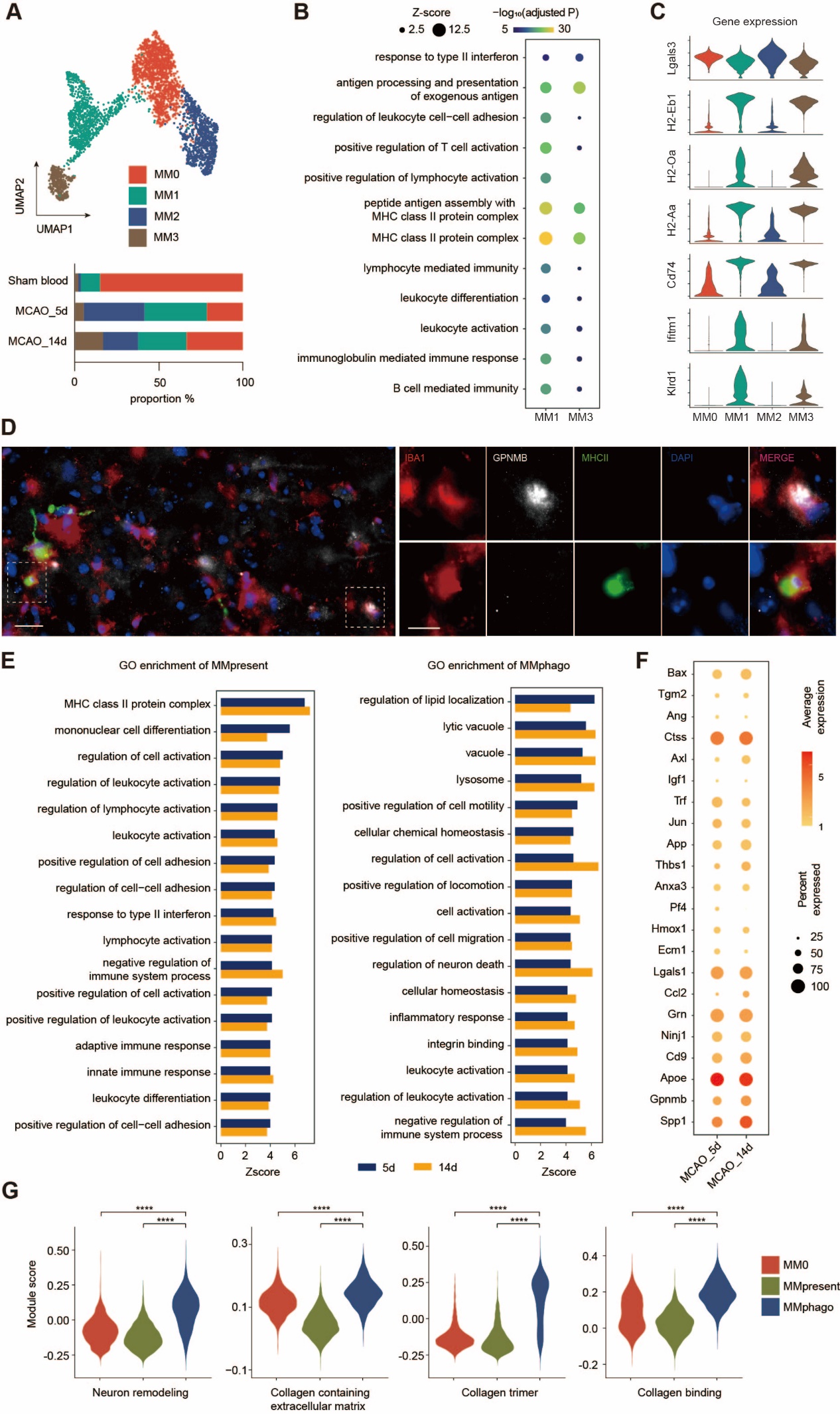
**

**Figure S2. Functions of different MM subsets and the pro-repair effects of MMphago.** (A) UMAP plot of all MM after unsupervised clustering (up) and stacked bar plot of cell proportion of different groups (down). (B) Dot plot displaying representative functional terms of MM1 and MM3 by GO enrichment based on Z-score and significance (-log_10_[adjusted P-value]). (C) Violin plots visualizing the marker gene in each cluster. (D) Representative images of GPNMB+ and MHCII+ MM immunofluorescence staining in the ischemic hemisphere on 14d after MCAO. Scale bars, 20 μm (low magnification) and 10 μm (high magnification). (E) Bar plot showing representative activated (Z-score ≥ 2) GO terms of biological process in MMpresent or MMphago, comparing the activation state on 5d and 14d after tMCAO. (F) Dot plot displaying the expression level of reparative genes in MMphago on 5d and 14d. (G) Violin plot displaying module scores of reparative functions. ****P-value < 0.0001 by the Wilcoxon Rank Sum test.


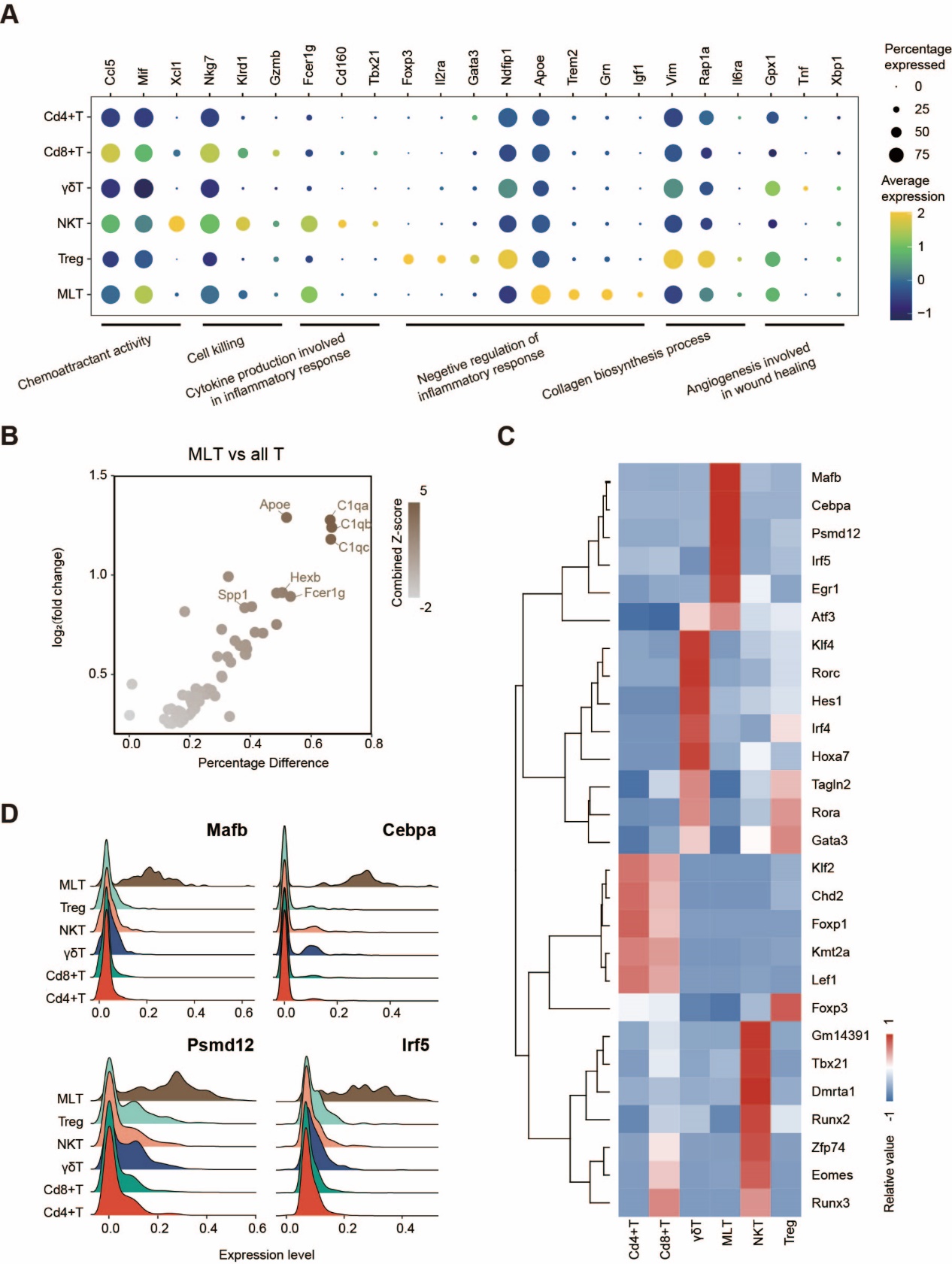


**Figure S3. The features of T cell subsets. (A)** Dot plot showing the expression level of functional genes in each cluster, related to Figure 3C. **(B)** Scatter plot showing the DEGs (log_2_(fold change) >0.25) of MLT compared to other T cells, with the percentage difference (defined as a percentage of the expression in MLT – in other T cells) along the y-axis and log_2_(fold change) along the x-axis. The combined Z-score of percentage difference and log_2_(fold change) was shown in the color scale. **(C)** Heatmap showing specific transcription factors of each cluster, with color scale representing relative expression. **(D)** Ridge plot showing the expression level of MLT-specific transcription factors measured by AUC score.


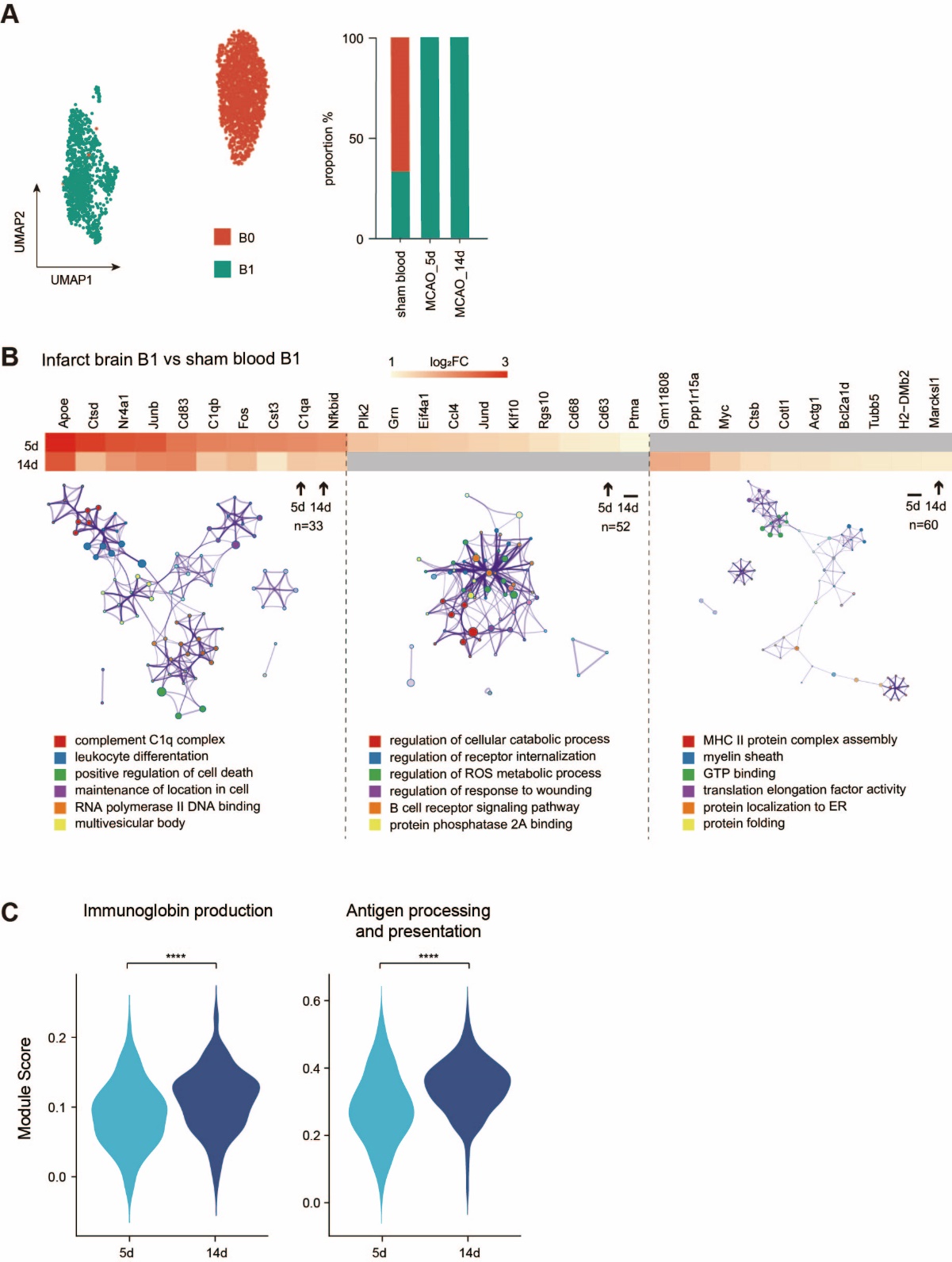


**Figure S4. Post-stroke dynamics of B lymphocytes. (A)** UMAP plot of B cells from sham blood and ischemic mouse brains after MCAO, with each color representing one subcluster (left). And stacked bar plot showing the proportion of the two clusters under each condition (right). **(B)** Heatmap showing the scaled log_2_(fold change) of DEGs of B cells in the brain compared to blood on 5d and 14d (up). And Metascape enrichment analysis for DEGs (down), with each node indicating a term. Clustering was made based on similarity (similarity > 0.3). Results were divided into three groups that included DEGs significantly upregulated on both 5d and 14d (left), only on 5d (middle), and only on 14d (right). **(C)** Violin plot displaying module scores of B cell functions and comparison between 5d and 14d. ****P-value < 0.0001 by the Wilcoxon Rank Sum test.

**
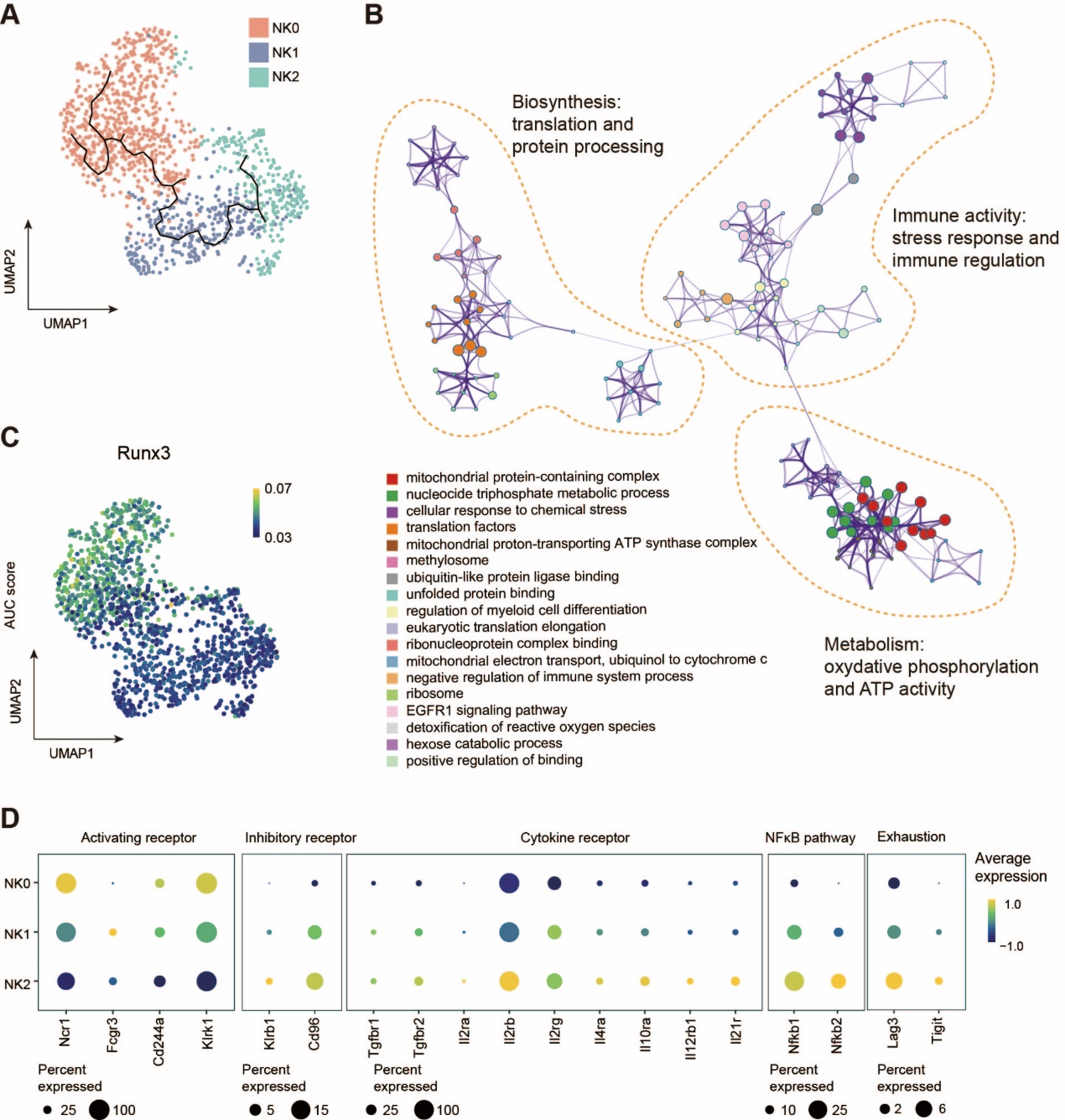
**

**Figure S5. NK cells functional changes and related genes. (A)** Pseudo-time trajectory of the differentiation of NK cells analyzed with Monocle3, showing a predicted shift from NK0 to NK2 based on acquired cellular trajectory. **(B)** Metascape enrichment analysis for DEGs of NK2 versus NK0, with each node indicating a term. **(C)** Feature plot displaying the expression level of Runx3 measured by AUC score. **(D)** Dot plot showing the expression level of functional genes in each NK cluster.

**
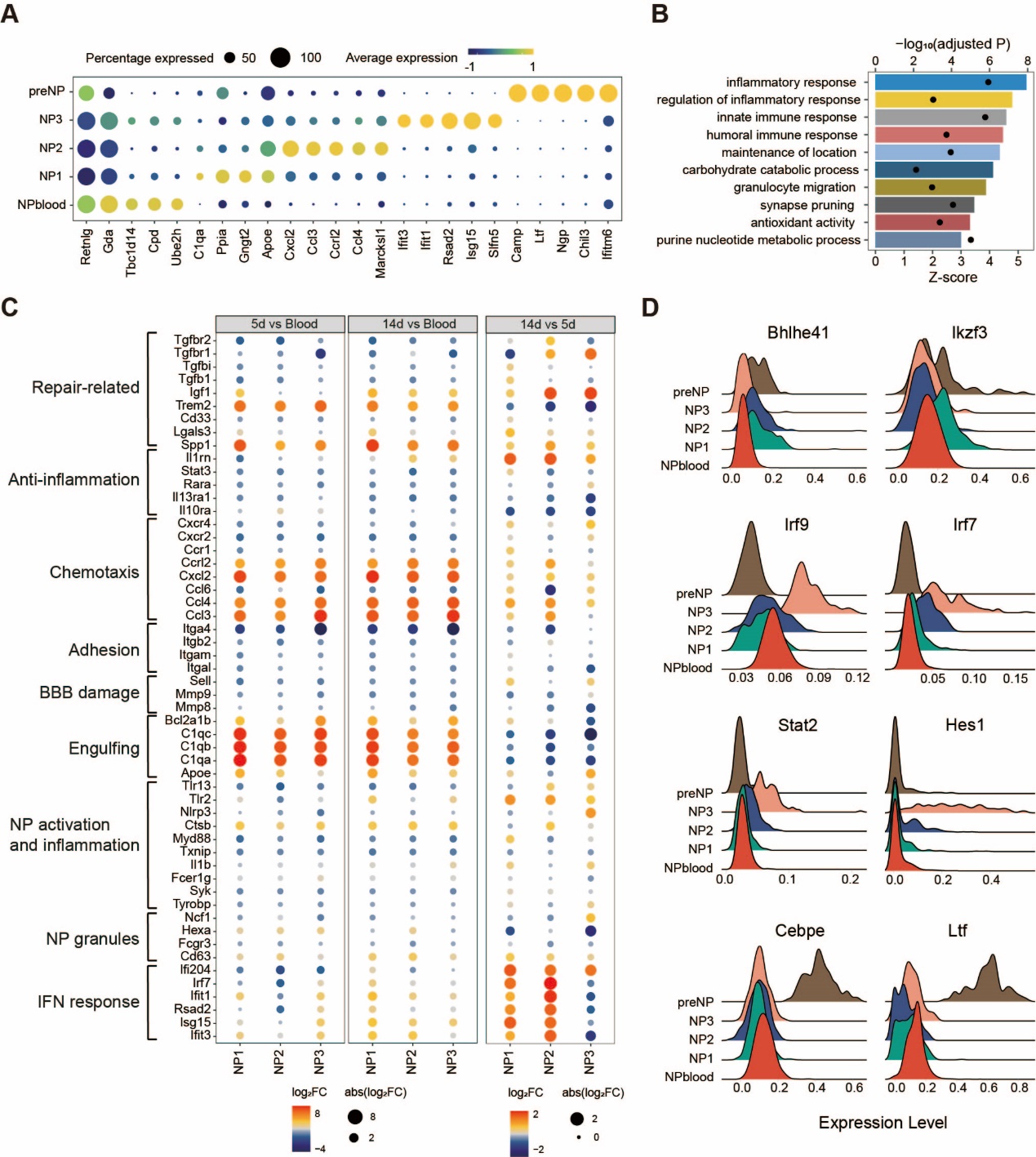
**

**Figure S6. Neutrophils exerted inflammatory and chemotaxis functions in the ischemic brain. (A)** Dot plot of marker genes for the five NP subclusters. **(B)** Bar plot showing the major upregulated GO terms of preNP, based on Z-score (bar) and significance (-log_10_[adjusted P-value]) (dot). **(C)** Bubble plot displaying a comparison of NP functional genes among different groups. **(D)** Ridge plot showing the expression level of NP1-, NP3-, and preNP-specific transcription factors measured by AUC score.
